# Putting Voluntary, Charity, and Social Enterprise (VCSE) Organisations at the Heart of Research: Creating a Northern Ireland VCSE Research Directory

**DOI:** 10.23889/ijpds.v11i1.3361

**Published:** 2026-05-14

**Authors:** Eric Spikol, Enya Redican, Orla McBride, Jamie Murphy, Mark Shevlin

**Affiliations:** 1 Ulster University, School of Psychology, Cromore Rd, Coleraine BT52 1SA, Northern Ireland, UK

**Keywords:** VCSE, not-for-profit sector, voluntary organisations, charity organisations, mapping exercise, Northern Ireland

## Abstract

**Introduction:**

Voluntary, community, and social enterprise (VCSE) organisations provide vital public services, support, and information while promoting and sustaining community cohesion. These organisations should be at the centre of research as they are uniquely placed to identify critical evidence gaps and to realise the full potential of research findings.

**Objective:**

A mapping exercise was undertaken to identify all VCSEs in Northern Ireland (NI), explore categories of organisational remit, identify key beneficiary and underserved populations, and to create a searchable database of NI VCSEs.

**Methods:**

Publicly available descriptive data provided by NI VCSEs were taken from not-for-profit sector administrative and regulatory organisation websites, and were then cleaned, categorised, and centralised in a directory tool.

**Results:**

In total, 40 primary remit categories and six unique secondary remit categories were identified, with N=17 categories covering distinct or specific beneficiary populations, N=3 having a non-human remit, and N=20 categories working on a community or general population level. Locational analysis showed the county-level distribution of VCSEs to be roughly proportional to the population percentages of each county.

**Conclusions:**

Compiling and summarising the NI VCSE landscape has provided critical insights into trends within the sector and facilitated the creation of a centralised, accessible, and searchable directory of all VCSE organisations in NI the VCSE-Finder NI, a freely available tool intended to facilitate engagement and networking between researchers and VCSEs.

## Introduction

Voluntary, community, and social enterprise (VCSE) organisations, often referred to collectively as the ‘not-for-profit sector’, are “...any organisation (incorporated or not) working with a social purpose” [1], regardless of size, remit, or operational level. Many VCSEs serve a charitable purpose and are registered as charities. VCSEs provide vital public services, support, and information while promoting and sustaining community cohesion. They are often a lifeline for vulnerable populations who may fall through administrative cracks, particularly in regard to health/mental health advocacy and support [2–4], or have needs which more traditional services cannot address, including social engagement and community interaction [5]. Our position in this paper is that VCSEs should be at the centre of research endeavours because they are uniquely placed to identify critical evidence gaps and to realise the full potential of research findings through improved resource allocation and ability to tailor and target services.

To secure and/or retain funding, VCSEs must regularly provide a significant amount of evidence to demonstrate the positive effect that their endeavours have had on the beneficiary population and society in general. This evidence can take many forms including supporting statistics relating to the population they support, economic evaluation of outcomes of service provision, as well as a comprehensive documentation of impact [6]. VCSEs therefore depend heavily on being able to source and/or produce high-quality evidence from high-quality data [7, 8]. However, VCSE operational budgets commonly prioritise provision of services to their beneficiaries, with less resources available to devote to such research, leading to increased reliance on lower quality evidence [9, 10]. Academic researchers often provide the necessary skills to generate such evidence, by working collaboratively with VCSEs; indeed, such collaborations are reciprocally beneficial as they can also demonstrate research ‘impact’ [11].

There have been initiatives to develop and support more academic researcher and VCSE collaboration, as networks have been established in some council areas in England including Greater Manchester [12] and North and East Cumbria [13]. These projects put the VCSE sector at the heart of research, giving organisations the opportunity to shape and guide the research using their ground-level knowledge of their beneficiary population’s needs [14] and their extensive experience of building relationships and trust with vulnerable or ‘hard to reach’ populations [15]. There have also been successful academic-VCSE collaborations in Northern Ireland (NI), a 6-county region of the United Kingdom (UK), and projects developed to encourage such work [16]. To facilitate these projects gaining traction and running smoothly, the number and remit of organisations and groups that comprise the VCSE sector needs to be understood. Currently however, no centralised, accessible, searchable directory of all VCSE organisations exists here. and so if academic-VCSE collaborations are to be encouraged and supported, such a resource is required. We propose that it is a necessary prerequisite to map the VCSE landscape in NI in order to better understand the sector and its needs.

To realise this, this paper was devised to address the following aims: 1) identify, collate, organise, and make accessible descriptive and contact information for all VCSE organisations in NI along multiple institutional and organisational metrics (e.g., location, size), 2) explore categories of organisational remit, 3) identify key beneficiary populations as well as underserved populations, 4) identify organisations for potential participation in proof-of-concept testing for a planned research engagement networking tool based on remit and beneficiary population, and 5) create a searchable database of VCSE organisations in NI.

## Methodology

### Study Procedure

This study aimed to create a directory of active VCSE or not-for-profit sector organisations in NI and further explore the VCSE landscape. UK-wide organisations were also included, provided they were operating from a branch or office located in NI. Data was gathered from VCSE ‘umbrella’ organisations and independently through web searches during September and October 2024, using the PRISMA systematic search framework [17]. Ethical approval was not required as no participants were involved and all data was sourced and compiled from publicly available information and resources.

### Search Strategy and Sources

**Figure 1:**
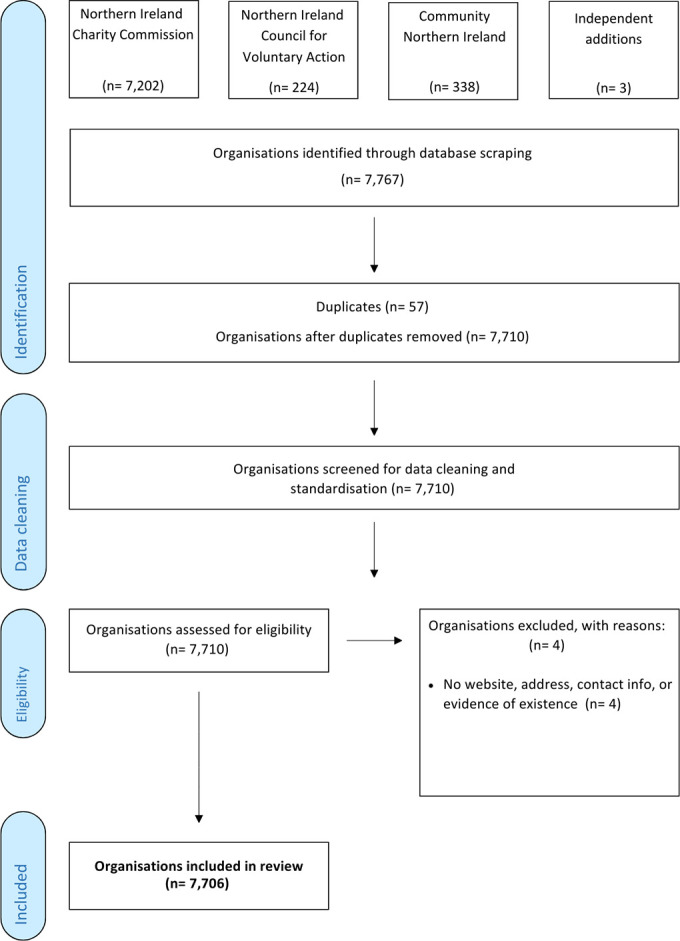
PRISMA Flow Diagram for Search and Identification of VCSE Organisations in Northern Ireland

Publicly available information for VCSE organisations operating (fully or partially) in NI was the target of this systematic search (Figure 1). As these were all centrally located in regulatory and administrative online repositories, no formal search strings were used. The full NI Charity Commission registry (*n* = 7,202) was available for download as a single file from the website (https://www.charitycommissionni.org.uk), while data was manually scraped from websites for the ‘umbrella organisations’ including the NI Council for Voluntary Action (NICVA; https://www.nicva.org; *n* = 224) and Community NI (https://www.communityni.org; *n* = 338). Independent web searches returned an additional *n* = 3 results.

### Data Extraction and Cleaning

Data extracted during manual data-scraping included all information posted about each organisation on its respective page through the NICVA and Community NI website, while data was taken directly from the websites of the *n* = 3 independently sourced results (Table 1). Data taken from the NI Charity Commission registry included these fields and some additional information describing charity-relevant aspects (e.g., charity registration number, date, and status, type of governing document, etc.) of the organisation. ‘Listings’ for organisations were not always complete, with many not displaying full contact information or a website. Prior to data cleaning, 57 duplicate results were removed. Additional data cleaning and web searches were performed to fill in as much information as possible. During data cleaning, *n* = 4 results were removed for having no website, physical address, contact information (email address or phone number), or any evidence of organisational existence other than registry, leaving a total of N=7,706 organisations included in the review.

**Table 1:** Data Extraction Categories by Data Source

	**NI Charity Commission**	**NICVA**	**Community NI**
Organisation name	X	X	X
Charity registration #	X		
Date registered	X		
Status	X		
Date for financial year ending	X		
Public address	X	X	X
Constituency		X	
Website	X	X	X
Contact name			X
Contact email	X	X	X
Telephone #	X	X	X
Charity function (by category)	X		
Beneficiary population (by category)	X		
Charity method (by category)	X		
Remit	X	X	X
Other name	X		
Type of governing document	X		
Number of employed staff	X		
Number of volunteers	X		

### Data Synthesis & Directory Development

Narrative synthesis was used to describe the exploring and mapping of the VCSE landscape in NI. The organisations were described in terms of remit, beneficiary population, location, and other metrics (where available). The raw data was explored using Microsoft Excel [18]. After data cleaning, the multi-tab spreadsheet was organised along several categorial metrics and formatting was standardised. Additional derived information, such as topic ‘tags’ were added to enable and enhance search functionality.

## Results

### Organisation Categorisation

After the raw data was imported into Excel, each organisation was assigned a primary subject category based on their remit and beneficiary population (Table 2) with the application of unique secondary categories for larger or distinct subgroups within a category. These included ‘parent-teacher association’ (association/union), ‘addiction’ (Mental health), ‘worship community’ (Religious), ‘orphans’ and ‘scouts’ (Youth), and ‘centre/hall’ (multiple). Some organisations received a secondary category based on specialisation within their primary category, for example a secondary category of ‘heritage’ within School/playgroup for Irish language schools or ‘disease/disability’ within Sport for sport teams/leagues comprised of individuals with specific health conditions or disabilities.

**Table 2:** Identified VCSE Organisations in Northern Ireland in 2025 by Remit Category

**Primary category**	**Secondary category**	**N=(%)**
Advice		**21 (<01%)**
	*Poverty*	*1 (4.7%)*
Age/ageing		**139 (1.8%)**
Agriculture		**20 (<1%)**
	*Training/education*	*2 (10%)*
Animals		**61 (<1%)**
Armed Forces		**36 (<1%)**
Arts		**417 (5.4%)**
	*Age/ageing*	*2 (<1%)*
	*Armed Forces*	*1 (<1%)*
	*Disease/disability*	*8 (1.9%)*
	*Heritage*	*42 (10.1%)*
	*LGBTQ+*	*2 (<1%)*
	*Men*	*1 (<1%)*
	*Mental health*	*2 (<1%)*
	*Minority*	*5 (1.2%)*
	*Peace/diversity*	*5 (1.2%)*
	*Religious*	*5 (1.2%)*
	*Youth*	*21 (5%)*
Association/Union		**450 (5.8%)**
	*Parent-teacher association*	*419 (93%)*
Bereavement		**10 (<1%)**
Carers		**16 (<1%)**
	*Disease/disability*	*4 (25%)*
	*Mental health*	*1 (6%)*
Community development		**1,127 (14.6%)**
	*Centre/hall*	*78 (7%)*
	*Health*	*1 (<1%)*
	*Housing*	*1 (<1%)*
Crime		**17 (<1%)**
	*Arts*	*1 (5.9%)*
	*Training/education*	*2 (11.8%)*
Disease/Disability		**436 (5.6%)**
	*Minority*	*1 (<1%)*
	*Religious*	*4 (<1%)*
	*Training/education*	*9 (2.1%)*
	*Women*	*2 (<1%)*
	*Youth*	*36 (8.2%)*
Environmental		**58 (<1%)**
	*Heritage*	*1 (1.7%)*
	*Justice*	*1 (1.7%)*
	*Training/education*	*5 (8.6%)*
Family		**41 (<1%)**
	*Centre/hall*	*22 (53.6%)*
Foreign beneficiary		**191 (2.5%)**
	*Religious*	*64 (33.5%)*
	*Youth*	*26 (13.6%)*
Health		**77 (1%)**
	*Sport*	*1 (1.3%)*
	*Youth*	*3 (4%)*
Heritage		**230 (3%)**
	*Centre/hall*	*3 (1.3%)*
Housing		**31 (<1%)**
	*Age/ageing*	*2 (6.4%)*
	*Disease/disability*	*2 (6.4%)*
Justice		**15 (<1%)**
	*Youth*	*1 (6.6%)*
LGBTQ+		**15 (<1%)**
Media		**13 (<1%)**
	*Heritage*	*1 (7.7%)*
	*Religious*	*3 (23%)*
	*Youth*	*1 (7.7%)*
Memorial trust		**91 (1.2%)**
	*Heritage*	*1 (1.1%)*
	*Peace/diversity*	*1 (1.1%)*
	*Religious*	*11 (12.1%)*
	*Sport*	*4 (4.4%)*
	*Training/education*	*5 (5.5%)*
Men		**42 (<1%)**
	*Age/ageing*	*2 (4.7%)*
	*Housing*	*1 (2.4%)*
	*Religious*	*1 (2.4%)*
Mental health		**141 (1.8%)**
	*Addiction*	*22 (15.6%)*
	*Centre/hall*	*2 (1.4%)*
	*Religious*	*1 (<1%)*
	*Women*	*1 (<1%)*
	*Youth*	*9 (6.4%)*
Minority		**81 (1.1%)**
	*Age/ageing*	*1 (1.2%)*
	*Heritage*	*47 (58%)*
	*Youth*	*1 (1.2%)*
NI conflict		**14 (<1%)**
	*Poverty*	*1 (4.3%)*
Peace/Diversity		**70 (<1%)**
	*Centre/hall*	*4 (7.1%)*
Poverty		**69 (<1%)**
	*Religious*	*5 (7.2%)*
Religious		**1,675 (21.7%)**
	*Centre/hall*	*41 (2.4%)*
	*Family*	*1 (<1%)*
	*Housing*	*1 (<1%)*
	*Justice*	*1 (<1%)*
	*LGBTQ+*	*1 (<1%)*
	*Training/education*	*4 (<1%)*
	*Worship community*	*1,254 (74.9%)*
	*Youth*	*21 (1.2%)*
Rescue/Response		**37 (<1%)**
School trust		**22 (<1%)**
School/Playgroup		**358 (4.6%)**
	*Disease/disability*	*1 (<1%)*
	*Heritage*	*18 (5%)*
Social club		**51 (<1%)**
Social welfare		**83 (1.1%)**
Sport		**264 (3.4%)**
	*Centre/hall*	*3 (1.1%)*
	*Disease/disability*	*26 (9.8%)*
	*Heritage*	*6 (2.2%)*
	*Mental health*	*2 (<1%)*
	*Minority*	*3 (1.1%)*
	*Religious*	*4 (1.5%)*
	*Women*	*2 (1%)*
	*Youth*	*26 (9.8%)*
Training/education		**130 (1.7%)**
	*Centre/hall*	*3 (2.3%)*
	*Youth*	*13 (10%)*
Transport		**24 (<1%)**
Victim services		**43 (<1%)**
	*Men*	*2 (4.6%)*
	*NI Conflict*	*9 (20.9%)*
	*Training/education*	*1 (2.3%)*
	*Women*	*15 (34.9%)*
Women		**84 (1.1%)**
	*Centre/hall*	*14 (16.6%)*
	*Minority*	*5 (5.9%)*
	*Training/education*	*2 (2.4%)*
Youth		**1,006 (13%)**
	*Centre/hall*	*29 (2.9%)*
	*Heritage*	*1 (<1%)*
	*Housing*	*2 (<1%)*
	*Orphans*	*9 (<1%)*
	*Peace/diversity*	*2 (<1%)*
	*Scouts*	*797 (79.2%)*
**TOTAL**		**7,706 (100%)**

In total, 40 primary categories and 6 unique secondary categories were created to describe the range in remit and beneficiary populations in the VCSE sector. Of these, 17 categories covered distinct or specific beneficiary populations (Age/Ageing, Carers, Disease/Disability, etc.), 3 categories had a non-human specific remit (Agriculture, Animals, and Environmental), and the remaining 20 categories worked on a community or general population level (Arts, Community Development, School/Playgroup, etc.).

**Figure 2:**
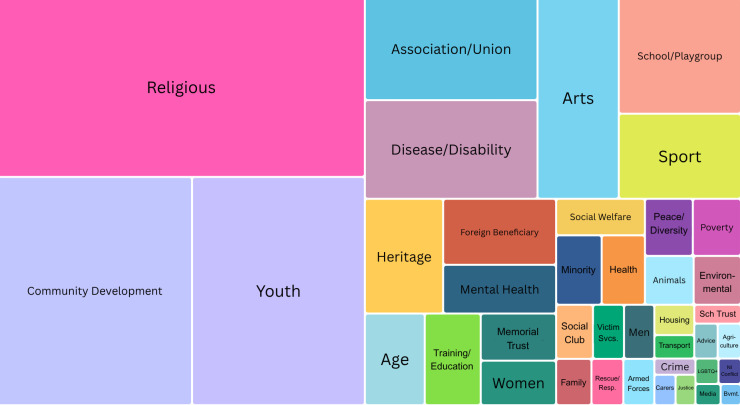
Tree Map* of the NI VCSE Sector in 2025

Religious, Community Development, and Youth were the largest categories, together comprising 49.4% of VCSE organisations in NI, with a further 25% being association/Union, Disease/Disability, Arts, School/Playgroup, and Sport, and the remaining 25% consisting of 32 smaller categories (Figure 2). It should be noted that 74.9% of religious organisations were Worship Communities (16.3% of the NI total) and 79.2% of Youth organisations were Scout groups (10.3% of the NI total). A semantic analysis of organisation names shows a majority featuring religious terms and place names.

* the rectangle for each category is proportional to the category size

**Figure 3:**
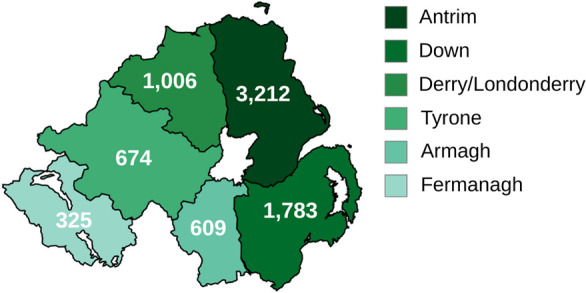
NI VCSE Organisation Count by County

A locational analysis by postal address (Figure 3) showed the county-level distribution of VCSEs, with 42.2% in Co. Antrim, 23.4% in Co. Down, 13.2% in Co. Derry/Londonderry, 8.9% in Co. Tyrone, 8% in Co. Armagh, and 4.3% in Co. Fermanagh. These percentages are roughly proportional to the population percentages of each county per Census 2021; Co. Antrim (34.22%), Co. Down (29.07%), Co. Derry/Londonderry (13.25%), Co. Armagh (10.21%), Co. Tyrone (9.91%), and Co. Fermanagh (3.34%). A total of *n* = 97 organisations were excluded from this analysis; *n* = 66 provided no postal address with their registration and *n* = 31 were UK-wide organisations providing services in NI but located in Great Britain. It should be noted that a majority of scouting organisations (*n* = 478, 60%) were registered through a headquarter address in Co. Down, accounting for 26.8% of that county’s VCSEs. Additionally, a majority of VCSEs in Co. Antrim (*n* = 1,771, 55.1%) were listed in NI’s capital city, Belfast.

Table 3 shows a breakdown of VCSEs categories by county with the percentage distribution again roughly concordant with each county population percentages, with the notable exception of Youth VCSEs in Co. Down as a result of scouting groups from across NI using the headquarters in Co. Down as their registered postal address.

**Table 3:** VCSE Organisation Category by NI County, 2025

	**Antrim N(%)**	**Down N(%)**	**Derry/L’D N(%)**	**Tyrone N(%)**	**Armagh N(%)**	**Fermanagh N(%)**
Advice	8 (38.1%)	5 (23.9%)	2 (9.5%)	2 (9.5%)	2 (9.5%)	2 (9.5%)
Age/ageing	50 (36%)	25 (18%)	20 (14.4%)	22 (15.8%)	15 (10.8%)	7(5%)
Agriculture	9 (47.4%)	4 (21%)	2 (10.5%)	1 (5.3%)	2 (10.5)	1 (5.3%)
Animals	25 (41.7%)	13 (21.7%)	9 (15%)	5 (8.4%)	4 (6.6%)	4 (6.6%)
Armed Forces	15 (44.1%)	6 (17.7%)	2 (5.9%)	4 (11.7%)	4 (11.7%)	3 (8.9%)
Arts	202 (50.3%)	72 (18%)	55 (13.7%)	24 (6%)	32 (8%)	16 (4%)
Association/union	184 (41%)	102 (22.6%)	63 (14%)	40 (8.9%)	30 (6.6%)	31 (6.9%)
Bereavement	5 (55.6%)	2 (22.2%)	1 (11.1%)	0 (0%)	1 (11.1%)	0 (0%)
Carers	7 (43.7%)	2 (12.5%)	0 (0%)	1 (6.2%)	3 (18.8%)	3 (18.8%)
Community dev.	428 (38.3%)	198 (17.7%)	206 (18.4%)	136 (12.1%)	85 (7.6%)	66 (5.9%)
Crime	10 (58.9%)	1 (5.9%)	4 (23.4%)	1 (5.9%)	1 (5.9%)	0 (0%)
Disease/disability	184 (43.2%)	101 (23.7%)	50 (11.7%)	43 (10.1%)	31 (7.3%)	17 (4%)
Environmental	32 (56.2%)	8 (14%)	4 (7%)	2 (3.5%)	5 (8.8%)	6 (10.5%)
Family	21 (53.8%)	5 (12.8%)	7 (18%)	3 (7.7%)	2 (5.1%)	1 (2.6%)
Foreign bene.	106 (57%)	40 (21.5%)	11 (6%)	9 (4.8%)	16 (8.6%)	4 (2.1%)
Health	46 (66.7%)	8 (11.6%)	7 (10.1%)	1 (1.5%)	4 (5.8%)	3 (4.3%)
Heritage	90 (39.3%)	47 (20.5%)	39 (17%)	21 (9.2%)	17 (7.4%)	15 (6.6%)
Housing	24 (77.5%)	3 (9.7%)	2 (6.4%)	1 (3.2%)	1 (3.2%)	0 (0%)
Justice	12 (80%)	2 (13.4%)	1 (6.6%)	0 (0%)	0 (0%)	0 (0%)
LGBTQ+	10 (66.6%)	1 (6.6%)	2 (13.4%)	2 (13.4%)	0 (0%)	0 (0%)
Media	6 (50%)	2 (16.6%)	3 (25%)	0 (0%)	1 (8.4%)	0 (0%)
Memorial trust	45 (51.1%)	17 (19.3%)	14 (16%)	7 (8%)	4 (4.5%)	1 (1.1%)
Men	10 (23.9%)	13 (31%)	6 (14.2%)	7 (16.6%)	4 (9.5%)	2 (4.8%)
Mental health	68 (50.4%)	20 (14.8%)	28 (20.7%)	8 (5.9%)	7 (5.2%)	4 (3%)
Minority	46 (58.2%)	6 (7.6%)	9 (11.4%)	4 (5.1%)	12 (15.2%)	2 (2.5%)
NI conflict	4 (30.7%)	2 (15.4%)	1 (7.7%)	2 (15.4%)	3 (23.1%)	1 (7.7%)
Peace/diversity	44 (63.9%)	7 (10.1%)	7 (10.1%)	4 (5.8%)	7 (10.1%)	0 (0%)
Poverty	42 (61.8%)	10 (14.7%)	8 (11.8%)	3 (4.4%)	4 (5.9%)	1 (1.4%)
Religious	704 (42.2%)	351 (21%)	214 (12.7%)	161 (10%)	170 (10.1%)	66 (4%)
Rescue/response	11 (30.5%)	9 (25%)	6 (16.7%)	7 (19.4%)	1 (2.8%)	2 (5.6%)
School trust	11 (50%)	3 (13.6%)	3 (13.6%)	3 (13.6%)	1 (4.6%)	1 (4.6%)
School/playgroup	110 (30.7%)	69 (19.3%)	65 (18.1%)	53 (14.8%)	31 (8.7%)	30 (8.4%)
Social club	20 (39.2%)	13 (25.5%)	7 (13.7%)	4 (7.8%)	6 (11.8%)	1 (2%)
Social welfare	52 (63.4%)	15 (18.3%)	4 (4.9%)	4 (4.9%)	6 (7.3%)	1 (1.2%)
Sport	122 (47%)	59 (22.7%)	37 (14.2%)	11 (4.2%)	27 (10.4%)	4 (1.5%)
Training/education	77 (62.1%)	17 (13.7%)	17 (13.7%)	6 (4.9%)	5 (4%)	2 (1.6%)
Transport	8 (33.4%)	3 (12.5%)	5 (20.8%)	2 (8.3%)	3 (12.5%)	3 (12.5%)
Victim services	22 (51.2%)	7 (16.2%)	6 (14%)	5 (11.6%)	2 (4.7%)	1 (2.3%)
Women	44 (53%)	10 (12%)	13 (15.7%)	11 (13.3%)	1 (1.2%)	4 (4.8%)
Youth	298 (29.7%)	505 (50.4%)	66 (6.6%)	54 (5.4%)	59 (5.9%)	20 (2%)

## Discussion

VCSE organisations are a central element for successful impact-generating research projects. To facilitate collaborations/partnership, researchers need to be familiar with the local VCSE landscape. We identified a gap in this knowledge base and developed a solution: the production of a detailed searchable directory for operating VCSE organisations in NI in 2025. Using a systematic methodology, our team developed a searchable directory by collecting and summarising information on these VCSE organisation, establishing discreet remit- and beneficiary-based categories, as well as locational and thematic mapping of the sector across all six counties in the region.

Results showed significant variation in remit and service provision from VCSEs in both specific and general beneficiary populations. VCSEs exist to respond to a societal need, thus this ‘map’ of the VCSE landscape offers an approximation of current societal need in NI. However, it is important to note that size is not always a determinant of larger need or greater reach. Additionally, categories such as Community Development and Religious (Worship Communities) are a significant percentage of the VCSE landscape as they serve local communities; there are likely to be multiple of these organisations within each community, and there are a significant number of small communities across NI. Size also cannot be taken as a measure of a met need in the beneficiary population. For example, Youth is the third largest category, largely due to scouting groups (79.2%), but youths remain a vulnerable and underserved population in NI [19].

Smaller remit categories are not necessarily indicative of a lower societal need as single organisations or a small number of organisations may operate sufficiently to negate the creation of additional ones. It is also possible that due to chronic underfunding and competition for limited resources, several remit categories are unable to grow to adequately serve their beneficiary populations. However, the increase of certain categories to meet need is vital in the case of emerging and/or growing beneficiary populations, such as non-native English speakers or those affected by rising cancer rates [20].

Counties with a higher population percentage generally showed higher numbers of VCSEs, as heavily populated areas typically require a greater infrastructure in all sectors. However, more sparsely populated rural areas in the UK often experience higher levels of deprivation along multiple indices [21, 22], which is also evident in NI [23]. Even in more urban areas, organisation count is not indicative of organisation success, as in the climate of a “funding crisis in the UK voluntary sector” [24] and increasing cuts [25, 26], VCSEs can run out of funding quickly, especially smaller/micro-organisations [27].

This only further underscores the need for high-quality evidence-based research which prioritises VCSE organisations while incentivising researcher/VCSE collaboration, and the first step in this process is the ability for VCSEs and researchers to find each other. In building the structure of an engagement networking directory with database functionality, the VCSE-Finder NI, our team focused on an interface which would give users search options to best fit their needs. This includes keyword searches by tag (“cancer”), direct name (“Carers NI”), primary/secondary category, location by county, city, and postal code stem, and beneficiary population. As popular search engine results are becoming increasingly irrelevant and inaccurate [28], the VCSE-Finder NI will allow users to find the organisation they’re searching for, or a range of organisations based on need, including accurate contact information and updated information where relevant.

**Figure 4:**
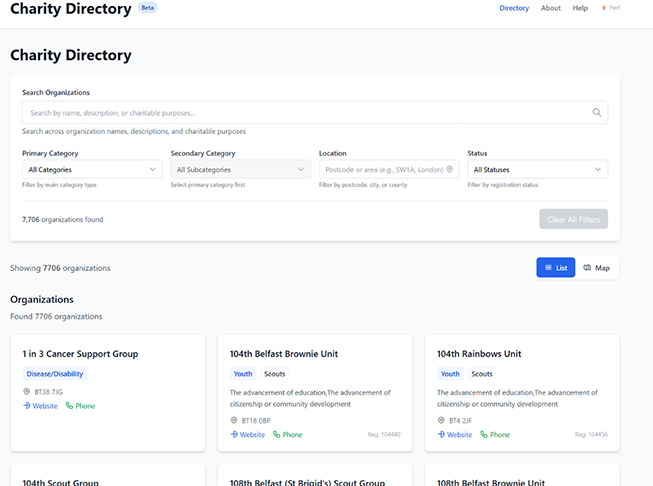
A Beta-version Example of the VCSE-Finder NI as a Web-Based Utility

Planning is underway to host the VCSE-Finder NI as a web-based utility through the [INSTITUTION REDACTED] website, with the above features, a user interface, mapping functionality, and multiple vector search options. Figure 4 below illustrates an example of what form this tool may take. This work, including processes for long-term directory maintenance and site and data management, is ongoing. Currently, the directory has been reposited for testing and proof-of-concept use using the Open Science Framework (OSF; https://osf.io). It is available for download and optional evaluation at https://osf.io/dprb9/. Our team encourages local NI users, users across the UK, and international users to download the VCSE-Finder NI for testing or as a model for similar tools. We look forward to partnering with the NI VCSE community, the statutory sector, and academics across all disciplines for a formal launch of the web-based version of the VCSE-Finder NI once complete.

### Strengths and limitations

There are several notable strengths associated with this exercise. This is the first comprehensive exploration into the VCSE sector in NI with a goal of categorically mapping the landscape by remit and beneficiary population. While the information summarised here is publicly available piecemeal, it has been collected, organised, and used to create a functional, up-to-date, and searchable directory to facilitate engagement, collaboration, and to boost inclusion of the NI VCSE sector in publicly beneficial research. The mapping process and auditing methodology described in this paper can serve as an exemplar for future UK regional or national research engagement networks, but also as a model for international replication.

The results here must be considered in light of the limitations of this mapping exercise. Data was taken from the public listings of the three main umbrella VCSE administrative organisations in NI and could not include organisations which have not registered or taken on membership with these organisations. Legal standings of the organisations were not considered, as not all organisations functioned as or were required to be government-registered entities. Additionally, it is likely that some of the organisations mapped here have now closed or dissolved since their most recent membership filing/refiling or registration with Community NI, NICVA, or the NI Charity Commission.

## Conclusions

Compiling and summarising the VCSE sector in NI has enabled our team to ‘map’ and explore trends within the landscape of the sector. From this, a database engagement and networking tool, the VCSE-Finder NI, was created and made available for public use with the goal of centring the VCSE sector in collaborative research for the benefit of the NI population.

## Data Availability

All data used in this study are publicly available for use or download through the Open Science Framework at https://osf.io/dprb9/. There are no restrictions on these data.

## Abbreviations

NI:	Northern Ireland
NICVA:	Northern Ireland Council for Voluntary Action
PRISMA:	Preferred Reporting Items for Systematic reviews and Meta-Analyses
UK:	United Kingdom
VCSE:	Voluntary Community and Social Enterprise

## References

[1] Cabinet Office. Procurement policy note – reserving below threshold procurements. [Internet]. 2020 [cited 2025 Aug 29]. Available from: https://www.gov.uk/government/publications/procurement-policy-note-1120-reserving-below-threshold-procurements.

[2] Martikke S, Moxham C. Public sector commissioning: experiences of voluntary organizations delivering health and social services. *Int J Public Adm*. 2010; 33:790-799. 10.1080/01900692.2010.521230

[3] Baggott R, Jones K. The voluntary sector and health policy: The role of national level health consumer and patients’ organisations in the UK. *Soc Sci Med*. 2014. 123:202-209. 10.1016/j.socscimed.2014.07.016 25085720

[4] Newbigging K, Rees J, Ince R, et al. The contribution of the voluntary sector to mental health crisis care: a mixed-methods study. Southampton (UK): NIHR Journals Library. 2020. 10.3310/hsdr08290 32644326

[5] Scott S, McGowan V, Wildman J, et al. “I’ll meet you at our bench”: adaptation, innovation and resilience among VCSE organisations who supported marginalised and minoritised communities during the Covid-19 pandemic in Northern England – a qualitative focus group study. *BMC Health Serv Res*. 2024. 24:7. 10.1186/s12913-023-10435-5 38172856 PMC10765907

[6] McGovern P. Getting needed resources: Life in small VCSE organisations. In: Small voluntary organisations in the ’age of austerity’. London: Palgrave Pivot; 2017. p. 50, p. 54-55. 10.1057/978-1-137-52188-0_4

[7] Pharoah C, Chapman T, Choudhury R. *An insight into charity funding in the North East*. London: Garfield Weston Foundation; 2014.

[8] Chapman T, Mawson J, Robinson F, Wistow J. How to work effectively with the third sector. Durham: Institute for Local Governance; 2018.

[9] Jochum V, Stuart J, Paine AE, Sheaff R, Exworthy M. Creating capacity: Developing and sharing knowledge between VCSEs and health and care commissioners. [Internet]. 2023 [cited 2025 Aug 29]. Available from: https://www.bayes.citystgeorges.ac.uk/__data/assets/pdf_file/0007/761326/Health-and-care-commissioning-and-the-VCSE-sector-research-briefing-2-October-2023.pdf.

[10] Pivotal. Scoping work for tackling destitution, poverty and economic insecurity in Northern Ireland: A summary of findings. [Internet]. 2023 [cited 2025 Aug 29]. Available from: https://www.pivotalppf.org/cmsfiles/NewsEvents/20240506-JRF-NI-Scoping-Study—Findings-Summary.pdf.

[11] UK Research and Innovation. Defining impact. [Internet]. 2025 [cited 2025 Aug 29]. Available from: https://www.ukri.org/councils/esrc/impact-toolkit-for-economic-and-social-sciences/defining-impact/

[12] National Institute for Health and Care Research (NIHR). The Greater Manchester Research Engagement Network (REN). [Internet]. 2025 [cited 2025 Aug 29]. Available from: https://arc-gm.nihr.ac.uk/ren.

[13] Voluntary Organisations’ Network North East (VONNE). North East and North Cumbria VCSE partnership programme. [Internet]. 2025 [cited 2025 Aug 29]. Available from: https://www.vonne.org.uk/north-east-and-north-cumbria-vcse-partnership-programme.

[14] Locock L, Boaz A. Drawing straight lines along blurred boundaries: qualitative research, patient and public involvement in medical research, co-production and co-design. *Evid Policy*. 2019. 15(3):409-421. 10.1332/174426419X15552999451313

[15] Flanagan SM, Hancock B. ’Reaching the hard to reach’ - lessons learned from the VCS (voluntary and community sector). A qualitative study. *BMC Health Serv Res*. 2010. 10:92. 10.1186/1472-6963-10-92 20377850 PMC2856561

[16] UK Research and Innovation. Realising the potential of Census 2021 data in Northern Ireland: Engage, inform and train. [Internet]. 2024 [cited 2025 Aug 29]. Available from: https://gtr.ukri.org/projects?ref=ES%2FZ502856%2F1.

[17] Page MJ, McKenzie JE, Bossuyt PM, Boutron I, Hoffmann TC, Mulrow CD, et al. The PRISMA 2020 statement: an updated guideline for reporting systematic reviews. *BMJ*. 2021. 372:n71. 10.1136/bmj.n71 33782057 PMC8005924

[18] Microsoft Corporation. Excel. [Computer Software]. 2021.

[19] Education Authority Northern Ireland. Youth funding awards target critical youth needs. [Internet]. 2025 [cited 2025 Aug 29] .Available from: https://www.eani.org.uk/news/youth-funding-awards-target-critical-youth-needs.

[20] Connolly ML. Cancer: Number of diagnoses in NI 18-49 age bracket up 20%. *BBC*. [Internet]. 2024 Mar 25 [cited 2025 Aug 29]. Available from: https://www.bbc.com/news/uk-northern-ireland-68651767.

[21] Pateman T. Rural and urban areas: comparing lives using rural/urban classifications. *Reg Trend*. 2011. 43:11–86. 10.1057/rt.2011.2.

[22] McAreavey R, Brown DL. Comparative analysis of rural poverty and inequality in the UK and the US. *Palgrave Commun*. 2019. 5:120. 10.1057/s41599-019-0332-8.

[23] McCann S, Ryan AA, McKenna H. The challenges associated with providing community care for people with complex needs in rural areas: a qualitative investigation. *Health Soc Care Community*. 2005. 13:462-469. 10.1111/j.1365-2524.2005.00573.x 16048534

[24] Association of Charitable Foundations. CEO blog | Understanding the funding crisis in the UK voluntary sector. [Internet]. 2024 [cited 2025 Aug 29]. Available from: https://acf.org.uk/acf/ACF/Blog/2024/CEO_blog_funding_crisis.aspx.

[25] Northern Ireland Affairs Committee. The funding and delivery of public services in Northern Ireland. [Internet]. 2024 [cited 2025 Aug 29]. Available from: https://committees.parliament.uk/publications/44014/documents/218038/default/.

[26] National Council for Voluntary Organisations. 2025: The year of the ’big squeeze’. [Internet]. 2025 [cited 2025 Aug 29]. Available from: https://www.ncvo.org.uk/news-and-insights/news-index/the-road-ahead-2025/challenges/.

[27] National Council for Voluntary Organisations. How many voluntary organisations are there?. [Internet]. 2023 [cited 2025 Aug 29]. Available from: https://www.ncvo.org.uk/news-and-insights/news-index/uk-civil-society-almanac-2023/profile/how-many-voluntary-organisations-are-there/.

[28] Guyton C. Google search might be getting worse - and AI threatens to ruin it entirely. *Tech Radar*. [Internet]. 2024 Jan 22 [cited 2025 Aug 29]. Available from: https://www.techradar.com/computing/search-engines/google-search-might-be-getting-worse-and-ai-threatens-to-ruin-it-entirely.

